# Targeting Reactive Oxygen Species in Atherosclerosis via Chinese Herbal Medicines

**DOI:** 10.1155/2022/1852330

**Published:** 2022-01-10

**Authors:** Leyi Zhang, Jiaqin Huang, Danli Zhang, Xiaojing Lei, Yan Ma, Yun Cao, Jingling Chang

**Affiliations:** ^1^University Postgraduate Education of Principles and Practice of Traditional Chinese Medicine, Medical University of Vienna, 1090 Vienna, Austria; ^2^Beijing University of Chinese Medicine, Beijing 100029, China; ^3^Department of Neurology, Dongzhimen Hospital, Beijing University of Chinese Medicine, Beijing 100700, China; ^4^Department of Pathophysiology and Allergy Research, Center for Pathophysiology, Infectiology and Immunology, Medical University of Vienna, 1090 Vienna, Austria

## Abstract

Cardio-cerebrovascular disease (CCVD) has become the leading cause of human mortality with the coming acceleration of global population aging. Atherosclerosis is among the most common pathological changes in CCVDs. It is also a multifactorial disorder; oxidative stress caused by excessive production of reactive oxygen species (ROS) has become an important mechanism of atherosclerosis. Chinese herbal medicine (CHM) is a major type of natural medicine that has made great contributions to human health. CHMs are increasingly used in the auxiliary clinical treatment of atherosclerosis. Although their mechanism of action is unclear, CHMs can exert a variety of antiatherosclerosis effects by regulating intracellular ROS. In this review, we discussed the mechanism of ROS regulation in atherosclerosis and analyzed the role of CHMs in the treatment of atherosclerosis via ROS.

## 1. Introduction

Cardio-cerebrovascular diseases (CCVD) refer to the ischemic and hemorrhagic diseases of the heart, brain, and body, caused by hyperlipidemia, blood viscosity, atherosclerosis, hypertension, among other factors [1]. CCVD have high incidence, high mortality rate, and high recurrence rate, which has become the first leading cause of noncommunicable diseases (NCD) deaths worldwide at over 50% and cause a considerable economic burden on global health [2]. The occurrence of cardiovascular and cerebrovascular diseases has many common risk factors, so they are closely related to each other. Atherosclerosis is among the most common pathological changes in CCVDs, it is considered to be a progressive inflammatory systemic disease that mainly affects the wall of large and medium arteries [3, 4]. Atherogenesis is a complicated course that concerns some mechanisms including endothelial dysfunction, vascular proliferation, apoptosis, matrix degradation, oxidative stress, inflammation, and thrombosis [3, 5]. Oxidative stress has been confirmed as a critical feature of the atherogenesis when detecting the oxidative modifications of lipids and proteins in vascular lesions since the 1950s [6, 7]. Reactive oxygen species (ROS) are important mediators and signal modifiers during various biological processes. It is widely defined that excessive ROS production outpacing the usable antioxidant systems results in oxidative stress [8], which can promote and increase the occurrence and development of atherosclerosis [9, 10]. As a consequence, antioxidant therapy is an ordinary way to atherosclerosis treatment. Angiotensin-converting enzyme inhibitors (ACEI), vitamins, angiotensin receptor antagonists, and stains can effectively mitigate oxidative stress [11]. Nevertheless, several issues have arisen during the application of antioxidants. Uncertain efficacy, higher cost and obvious side effects make antioxidative therapy unacceptable to most patients, which resulted in limited promotion and application. Bleys et al. [12] conducted a meta-analysis indicating that vitamin supplement therapy cannot prevent the atherosclerosis progression. Probucol was first listed as a lipid-lowering drug in the United States in 1977 and has been recognized as the most promising and effective first-line antioxidant in the treatment of atherosclerosis [13]. But a problem with probucol is that it prolongs the QT interval with proarrhythmic risk [14].

Hence, it is of great clinical significance to search for new therapies to treat the patients with atherosclerosis. Nowadays, Chinese herbal medicines (CHMs) have been applied for thousands of years with long experience and recognized as a typical representative of complementary and alternative medicine. Based on the holistic view of traditional Chinese medicine (TCM), it is feasible for CHMs to make the most of its advantages of imparting multiple approaches with multiple targets for the treatment of atherosclerosis [15]. Some scholars found out that CHMs could exhibit an antioxidant advantage. Especially, active compounds extracted from CHMs have therapeutic effects on oxidative stress in vivo and vitro, providing an approach for the development of antiatherosclerosis drugs. A great number of crude herbs, extracts, and isolated compounds have been screened for their antioxidant properties in vitro and in vivo [16, 17]. Meanwhile, accumulating evidence showed that well-designed clinical trials in the prevention and treatment of CCVDs based on the antioxidative therapy by CHMs acquire certain efficacy [18, 19]. Therefore, this article reviews the published literatures to investigate the mechanisms of oxidative stress under the effect of CHMs.

## 2. Molecular Mechanisms of Oxidative Stress in Atherosclerosis

### 2.1. Generation Mechanism of ROS

Many studies currently consider that excessive production of ROS can lead to the occurrence of oxidative stress, which plays a crucial role in the occurrence of atherosclerosis [20]. The principal ROS include hydroperoxyl, superoxide, peroxyl, and hydroxyl [21, 22]. As a highly active molecular substance, ROS is widely involved in redox reactions in the body [23]. Although being an important material basis for maintaining the stability of the vascular environment, overproduced ROS can also participate in the occurrence of vascular damage. Regarding the source, endogenous ROS generation mechanisms include NADPH oxidase (NOX), nitric oxide synthase (NOS), mitochondrial respiratory chain, and xanthine oxidase [24, 25].

Recent studies have found that NOX, as a membrane-bound enzyme complex, is extremely important in the process of promoting the production of oxygen free radicals. The subunit of NOX is the main source of ROS produced by vascular cells [26]. The membrane-bound enzymes of the NOX family transfer electrons from NADPH to O_2_, thereby generating superoxide radicals [27]. There are 7 kinds of NOX homologues that have been confirmed in humans, namely, membrane subunits NOX1-5 and DUOX1-2 (Dual Oxidase 1-2) [28], which has been discussed for their roles in atherosclerosis. Although NOX is present in the whole blood vessel wall cells, such as endothelial cells (EC), smooth muscle cells (SMCs), and fibroblasts, the expression of the subunits of the enzyme is different in these cells. NOX-1 and NOX-4 are mainly expressed in vascular smooth muscle cells [29, 30], while NOX-2 and NOX-4 in endothelial cells [31–33]. As shown in in vivo and in vitro studies, NOX-1 can regulate the development of atherosclerotic lesions by regulating the amount of collagen content in the neointimal space [34], macrophage infiltration [35], and migration of SMCs [36]. Similar to NOX-1, the deficiency of NOX-2 has also been confirmed to reduce the occurrence of atherosclerosis [37]. In contrast to NOX-1 and NOX-2, NOX-4 was found to have cardioprotective effects, which involves multiple mechanisms. This may be related to the hydrogen peroxide produced by NOX-4 instead of superoxide [38], and not react with NO [38, 39]. Besides, NOX-5 has also been shown to increase expression in atherosclerotic lesions [40], which may be related to the proliferation of EC and SMC [41, 42]. It can be seen from the above that the NOX enzymes in monocytes/macrophages and blood vessel wall cells are necessary for the development of atherosclerosis.

NO controls regulatory function such as neurotransmission or vascular tone. A mode of inaction of NO is the reaction with superoxide anion (O_2_^−^) to form the potent oxidant peroxynitrite (ONOO^−^), which can cause oxidative damage. NOS produce NO from L-arginine (L-Arg) in the presence of O_2_ and NADPH. The results of animal experiments revealed that a different NO synthase (NOS) plays an opposite role in atherosclerosis, including neuronal NOS (nNOS), inducible NOS (iNOS), and endothelial NOS (eNOS). Among them, eNOS is most closely related to the pathology and physiology of blood vessels and plays a protective role with nNOS in atherosclerotic diseases, whereas iNOS promotes the development of atherosclerotic [43]. It has been proved that NO released by eNOS with the assistance of tetrahydrobiopterin (BH4) can scavenge oxygen free radicals, inhibiting the expression of adhesion molecules, and promote the adhesion of lymphocyte [44–46]. But interestingly, previous studies have also found that overexpression of eNOS can accelerate the development of atherosclerosis in ApoE-KO mice [47]. Later, researchers upregulated BH4 synthesis in ApoE-KO/eNOS-Tg mice to reduce the effect of eNOS uncoupling, finding that can inhibit the occurrence of atherosclerotic lesions [48, 49]. The concentration of NO is related to the regulation of eNOS by BH4 in the blood vessels [50]. Therefore, the above contradiction may be caused by the consumption of BH4 when oxidative stress occurs, leading to uncoupling of eNOS. Moreover, in animal experiments, it is found that nNOS can dilate blood vessels and act synergistically with eNOS [51, 52], while the human studies have also shown that nNOS plays an important role in the regulation of vascular tone. On the contrary, iNOS is usually not expressed in the blood vessels under physiological conditions, but expressing when inflammation or oxidative stress occurs [53]. iNOS induces the production of excessive NO and promotes the increase of ROS, such as peroxynitrite (OONO-), which is the key to the development of atherosclerosis [54, 55]. It is worth noting that iNOS will combine BH4 to compete with eNOS, thereby reducing eNOS-mediated NO production [56].

As an important organelle of oxidative stress, the mitochondria can produce excessive ROS during hypoxia [57]. The mitochondria employ an intricate network of ROS scavenging system that coordinately work to mitigate oxidative stress [58]. In fact, atherosclerosis is closely related to mitochondrial dysfunction [59]. Dysfunctional mitochondria produce excessive amounts of ROS such superoxide (O_2_^−^), hydrogen peroxide (H_2_O_2_), and peroxynitrite (ONOO^−^). Experiments in ApoE-KO mice lacking the antioxidant system also found that the development of atherosclerosis and the increase of mitochondrial ROS both occurred, suggesting that the increase of ROS caused by mitochondrial dysfunction may play a role in atherosclerosis [60].

Apart from the above methods, xanthine oxidase (XO) can produce superoxide anions and hydrogen peroxide, which exists in endothelial cells and plasma [20]. And the level of XO was found to be increased in human atherosclerotic plaque [61]. Current research suggests that xanthine oxidase promotes the expression of LOX-1 and CD-36 in macrophages and SMCs, leading to an increase in ROS [62]. Moreover, some XO inhibitors, such as febuxostat [63], can prevent the development of atherosclerosis in ApoE-KO mice. Another population study also revealed that allopurinol can reduce the risk of coronary artery disease [64]. However, the specific role of XO in atherosclerosis remains to be further explored.

### 2.2. Effects of ROS on Blood Vessel Cells

**Figure 1:**
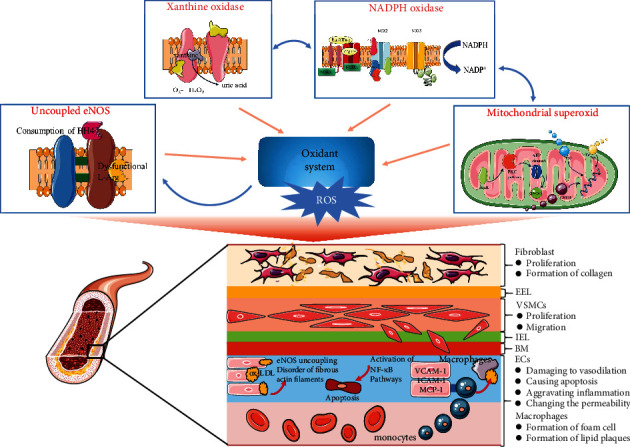
Oxidative stress mechanisms in atherosclerosis. ROS is the key to oxidative stress in atherosclerosis, derived from NADPH oxidase (NOX), nitric oxide synthase (NOS), mitochondrial respiratory chain, and xanthine oxidase. ROS promotes the occurrence and development of atherosclerosis by damaging the function and structure of vascular endothelial cells, smooth muscle cells, mononuclear macrophages, and fibroblasts.

As a key initiating factor of oxidative stress [65], the excessive increase of ROS production will cause an imbalance between oxidation and antioxidation, which can lead to the occurrence of oxidative stress [66]. Oxidative stress caused by excessive production of ROS can directly act on intracellular macromolecular substances and produce toxic effects. Furthermore, ROS or other oxidation products are used as signal transduction molecules to activate related pathways and damage the blood vessel cells, including endothelial cells (ECs) and vascular smooth muscle cells (VSMCs), ultimately promoting the onset of atherosclerosis [67]. The damage mechanism of oxidative stress in the vascular is shown in Figure 1.

Because of exposure to endogenous oxidative stress and direct continuous stimulation of oxidized lipids, vascular endothelial cells are mostly susceptible to peroxidation damage, which in turn affects their normal functions and structures [68]. ROS can damage vascular endothelial cells in the following ways: (1) damaging to endothelium-dependent vasodilation. NO is the main mediator of endothelium-dependent vasodilation [69]. Endothelial damage in the early stage of atherosclerosis leads to the oxidation of low-density lipoprotein cholesterol (LDL) in the subendothelial cavity to form oxidized LDL (ox-LDL) [7, 70], which can inhibit the activity of NOS in endothelial cells and accelerate the degradation of NO, thereby reducing biologically active NO and the bioavailability of NO, eventually causing endothelium-dependent vasodilation abnormalities. In addition, during oxidative stress, BH4, a cofactor of NOS, is oxidized and depleted by nitrogen peroxide, and eNOS is uncoupled, resulting in a decrease in NO production [71]. (2) Causing apoptosis of vascular endothelial cells. Previous studies have shown that endothelial cell apoptosis may play an important role in the erosion and rupture of atherosclerosis lipid plaques [72]. ROS has a regulatory effect on the apoptosis mechanism of endothelial cells. First, recent studies have found that ROS can directly activate NF-*κ*B, or indirectly through redox factor-1 (Ref-1). Being activated, NF-*κ*B translocates into the nucleus, combining with the apoptosis-related gene c-Myc to promote gene transcription and induce apoptosis [73]. In addition, ROS causes lipid peroxidation to destroy the mitochondrial inner membrane and regulates the exogenous and endogenous apoptotic pathways through activating caspase 3 and caspase 9, ultimately leading to endothelial cell apoptosis [74]. ROS can also activate the p38 pathway and c-Jun N-terminal kinase pathway to promote cell apoptosis [75]. (3) Inducing the expression of vascular endothelial cell adhesion molecules and aggravating inflammation. Adhesion of monocytes to the vascular endothelium is the initiation event of AS, in which adhesion molecules play a key role, such as vascular cell adhesion molecule-1 (VCAM-1) [76], intercellular adhesion molecule-1 (ICAM-1) [77], and monocyte chemoattractant protein-1 (MCP-1) [78]. As vascular endothelial cells are stimulated by ROS, the gene expression of these adhesion molecules is upregulated, which promotes the adhesion of monocytes and the release of inflammatory molecules, finally aggravating the inflammatory [79, 80]. (4) Changing the permeability of endothelial cells. Due to the existence of cytoskeleton, vascular endothelial cells can maintain cell morphology and regulate normal adhesion between cells. ox-LDL can induce the destruction, rupture, and disorder of fibrous actin filaments, which in turn leads to increased endothelial cell permeability. However, the increase in the intercellular space is more conducive for lipid components entering to the endothelium, which results the occurrence and development of atherosclerosis [81]. Furthermore, as an important influencing factor of vascular tension [82], the migration and proliferation of VSMCs are also involved in the pathogenesis of AS [83]. There have been a large number of early reports confirming that ROS can promote the proliferation of VCSMs [84–86]. In addition, ROS has also been shown to affect the proliferation of VSMCs by mediating hormones and growth factors. For instance, PPAR*γ* can reduce oxidative stress by upregulating UCP2 and ultimately inhibit the proliferation and migration of VSMCs [87]. Similarly, ROS regulates VSMC migration with several key mechanisms, including actin polymerization, focal adhesion kinase activation, and lamellipodia formation [88, 89]. As for the effect on monocytes and macrophages, the oxidative modification of LDL induced by ROS contributes to the adhesion of monocytes to endothelial cells and migration to the inner membrane to differentiate into macrophages. In the process of lipid peroxidation, the highly reactive oxygen metabolism intermediates produced in large quantities are combined with apolipoprotein B100, which prevents negative feedback metabolism, causing macrophages and VSMCs to take up a large amount of ox-LDL and become foam cells and lipid plaques [90].

In summary, ROS has multiple sources of generation [91], and the oxidative stress mediated by it can damage vascular cells from both structure and function, which is also an important pathological basis for atherosclerosis [92]. Therefore, it is of great clinical significance to use the ROS-mediated oxidative stress mechanism as a target to explore new methods for the treatment of atherosclerosis. As a commonly used clinical method of diseases, CHMs has great potential in the treatment of atherosclerosis [93]. Recently, more and more studies have been carried out on the oxidative stress mechanism of CHMs intervention in atherosclerosis. However, due to the diversity of CHMs and the characteristics of multiple targets, this type of research is diverse, but lacking of a systematic review. Based on the above phenomena, this article discusses the specific mechanisms of CHM intervention in atherosclerosis from three key sources of ROS, NOX, NOS, and mitochondrial dysfunction, in order to provide references for future research on the treatment of atherosclerosis with CHMs.

## 3. Herbal Medicine: Promising Therapeutic Agents for the Modulation of Oxidative Stress in Atherosclerosis

### 3.1. Prescriptions

**Figure 2:**
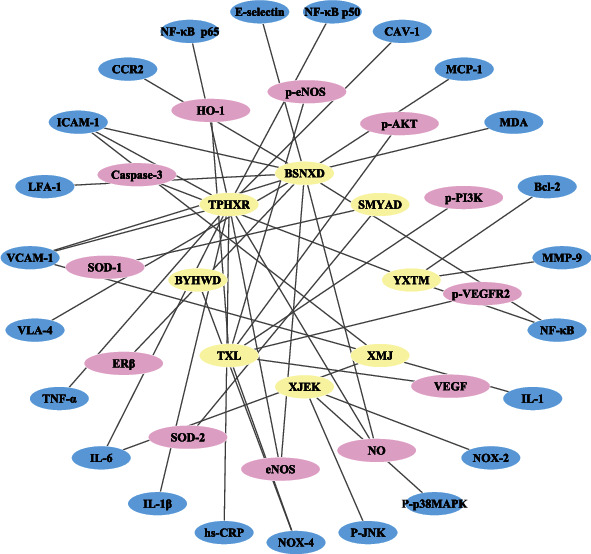
Network diagram of prescription targets in the modulation of oxidative stress in atherosclerosis (the yellow ellipses in the innermost circle are the prescriptions, the pink ellipses in the middle circle represent the upregulated targets, and the blue ellipses in the outside circle denote the downregulated targets).

Clinical studies have confirmed that TCM prescriptions exhibit significant antioxidative efficacy in the treatment of atherosclerosis. By measuring cell viability in vitro, Shen et al. examined the Buyang Huanwu decoction (BYHWD) played a vital role in protecting neurons against cerebral ischemic injury by downregulation of the expression of NOX-4 and reducing the production of ROS [94]. Guo et al. [95] found that Yixingtongmai (YXTM) could inhibit the expression of matrix metalloproteinase 9 (MMP-9), nuclear factor kappa-B (NF-*κ*B), and B cell lymphoma-2 (Bcl-2) and stimulate the activity of caspase 3, suggesting its effects in preventing the development and progression of atherosclerosis. Wang et al. [96] demonstrated that the herbal compound Tongxinluo (TXL) significantly increased myocardial capillary density and alleviated oxidative stress injury in heart failure by inhibiting the expression of NOX-4 and upregulating cardiac nitrite content and the protein expression of vascular endothelial growth factor (VEGF), p-VEGFR2, p-phosphoinositide 3-kinase (p-PI3K), p-AKT, (p-eNOS), andh oxygenase-1 (HO-1). Ren et al. [97] reveals that Si-Miao-Yong-An decoction (SMYAD) can apparently improve heart function through restoring the equilibrium of SOD and NOX-2, which significantly upregulated SOD-1 and SOD-2 mRNA expression levels and reduced NADP/NADPH ratio. Guo et al. [98] confirmed that Xin-Ji-Er-Kang (XJEK) had an antihypertensive and myocardial protective effect via reducing cardiac remodeling and improving endothelial dysfunction and oxidative stress. The expression of NOX-2, P c-Jun N-terminal kinase (JNK), and P-p38 mitogen-activated protein kinases (MAPK) was suppressed by XJEK therapy. Wang et al. [99] showed that the active components of Bu-Shen-Ning-Xin decoction (BSNXD) could increase the expression of estrogen receptor beta (ER*β*) in the HUVECs, reduce the production of malondialdehyde (MDA), upregulate the expression of eNOS, and promote the synthesis of NO. Yin et al. identified Xin-mai-jia (XMJ) could reduce serum levels of cytokines, including intercellular adhesion molecule-1 (ICAM-1), Vascular cell adhesion molecule-1 (VCAM-1), interleukin-1 (IL-1), and interleukin-6 (IL-6) [100]. Wen et al. showed that high and medium doses of Tiaopi Huxin recipe (TPHXR) decreased the expression of caveolin-1 (CAV-1), NF-*κ*B p50, NF-*κ*B p65, ICAM-1, VCAM-1, tumor necrosis factor-*α* (TNF-*α*), IL-6, and interleukin-1*β* (IL-1*β*) in the vasculature of ApoE(-/-) mice; decreased tumor TNF-*α*, IL-6, hypersensitive-CRP (hs-CRP), and IL-1*β* in mouse plasma; promoted eNOS phosphorylation; increased NO level; and improved endothelium-dependent vasodilation [101]. The specific mechanisms in vivo and in vitro of the above prescriptions are shown in Figure 2.

### 3.2. Chinese Herbal Compounds

In vitro and in vivo experiments also confirmed that herbs and its extract had significant effects in the treatment of atherosclerosis. Accumulating evidence that many CHMs have the effect of inhibiting NOXs to protect vascular endothelial cells is ontained. Dong et al. [102] demonstrated that icariin attenuated the proliferation and remodeling of cerebrovascular SMCs by inhibiting the activation of NOX-2-containing NOX, indicating that icariin may be a potential therapeutic agent for preventing the onset and progression of stroke. Puerarin, a major bioactive constituent of the Radix puerariae, can protect the cardiovascular system by the decrease in endogenous ROS production and NOX-2 expression. Xu et al. confirmed this conclusion [103]. To evaluate the preventive and curative effects of scutellarin on atherosclerosis in rats, Mo et al. [104] found that scutellarin prevented oxidative stress-induced vascular endothelial dysfunction and endothelial cell damage and hindered the process of atherosclerosis through antioxidant effects in vivo.

Aucubin (AU) is one of the active components of eucommia ulmoides. Li et al. found that in H_2_O_2_-induced SH-SY5Y cells, AU inhibits the expression of p-NF-*κ*B/NF-*κ*B by activating nuclear factor E2-related factor 2/hemo oxygenase-1 (NRF2/HO-1), downregulates MAPK and Bcl-2/Bax pathways, and significantly reduces the content of TNF-*α*, IL-6, and IL-1*β* and the rate of apoptosis, reduces the production of ROS and the content of MDA, and increases the content of glutathione (GSH) and the activity of SOD, and inhibits the expression of iNOS protein to reduce the production of NO [105]. Bergaptol (BER) is one of the constituents of Citrus aurantium Linn variant amara Engl (CAVA). In macrophages stimulated with lipopolysaccharides (LPSs), Shen et al. showed that BER could significantly inhibit the production of NO, IL-6, and TNF-*α*, and the gene expression of iNOS, IL-6, TNF-*α*, IL-1*β*, and COX-2. BER also blocked phosphorylation of MAPK phosphorylation and NF-*κ*B, which showed an inhibitory effect on JNK, p38, p65, inhibitor of NF-*κ*B (I*κ*B*α*) and I*κ*K*α*/*β* phosphorylation, and NF-*κ*B nuclear translocation [106]. Sundar et al. [107] showed that aqueous extract of P. sarmentosum leaves (AEPS) treatment successfully increased dimethylarginine dimethylaminohydrolase1 (DDAH1) mRNA expression, DDAH1 protein level, and DDAH1 activity in TNF-*α*-treated HUVEC and promoted eNOS production. Yang et al. showed that water extract of Korean Red Ginseng (KRG-WE) could reduce the production of NO, suppressed mRNA levels of iNOS, COX-2, and interferon-*β* (IFN-*β*) by inhibiting p38, JNK, and TANK binding kinase 1 (TBK1) and by subsequent inhibition of activating transcription factor 2 (ATF-2), cAMP-response element binding protein (CREB), and interferon regulatory factor 3 (IRF-3) activation [108]. Lian et al.'s research showed that, in the thoracic aortas of atherosclerotic rats, ginkgetin could reduce the mRNA and protein expressions of MMP-2, MMP-9, and iNOS in the thoracic aorta and increase the levels of NO and NOS in the serum and thoracic aorta [109]. Liu et al. proved that honokiol could downregulate TNF-*α*, IL-6, and IL-1*β*, decrease the level of ROS, increase the activity of superoxide dismutase, and significantly inhibit the level of NO, the expression of iNOS, and the abnormal activation of nuclear factor-*κ*B pathway in ApoE(-/-) mice [110]. Luo et al. found that total aralosides of Aralia elata (Miq) Seem (TASAESs) can significantly reduce the size of atherosclerotic plaque and the expression level of caspase 3 in aortic valve of ApoE(-/-) mice. TASAES can increase the viability of HUVECs cells, weaken the depolarization of mitochondrial membrane potential, promote endothelial cell apoptosis, and activate SIRT1/AMPK and AKT/eNOS signal pathways [111]. Zhou et al. showed that oligomeric proanthocyanidins from Rhodiola rosea (OPCRR) can significantly reduce the serum lipid profiles including total cholesterol, total triglycerides, low-density lipoprotein (LDL-C), and ox-LDL and increase HDL-C. It significantly increased SOD and glutathione peroxidase (GSH-Px) and decreased the content of malondialdehyde (MDA); reduced serum levels of TNF-*α*, IL-1*β*, IL-6, ICAM-1, and VCAM-1; increased the level of IL-10; significantly decreased serum NO; inhibited the expression of iNOS; and increased the expression of eNOS [112].

**Figure 3:**
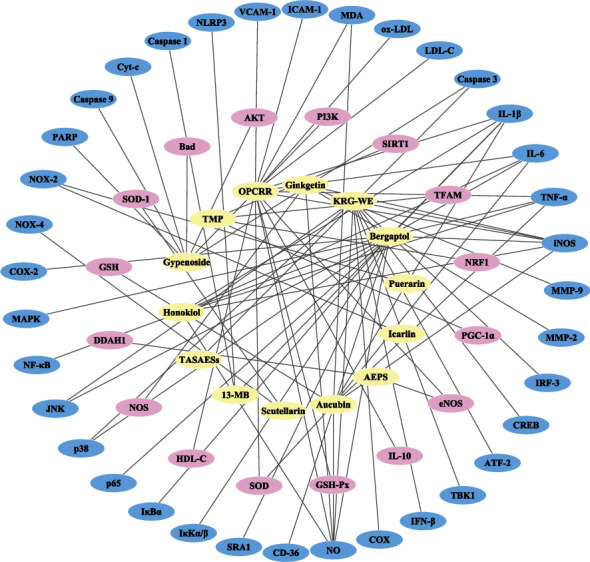
Network diagram of compounds in the modulation of oxidative stress in atherosclerosis (the yellow ellipses in the innermost circle are the compounds, the pink ellipses in the middle circle represent the upregulated targets, and the blue ellipses in the outside circle denote the downregulated targets).

The treatment of CHMs for atherosclerosis may also play an effect by regulating the function of mitochondria. Peng et al. [113] investigated the potential role of 13-methylberberine (13-MB) in atherosclerosis in vitro. The results affirmed that a retreatment with 13-MB markedly reduced the level of NOD-like receptor protein 3 (NLRP3), caspase 1, and IL-1*β* in H_2_O_2_-induced HUVECs. Xu et al. showed that tetramethylpyrazine (TMP) could protect the endothelium at the cell levels. TMP acts as an antioxidant in the mitochondria and improves mitochondrial dysfunction and is able to reverse high glucose-induced suppression of sirtuin-1 (SIRT1) and the biogenesis-related factors, including peroxisome proliferator-activated receptor gamma coactivator-1 alpha (PGC-1a), nuclear respiratory factor 1 (NRF1), and mitochondrial transcription factor A (TFAM) [114]. Song et al. [115] found that gypenoside significantly upregulated PI3K, AKT, and Bcl-xL/Bcl-2-associated death promoter (Bad) protein levels, and the expression of cytochrome C (Cyt-c), caspase 9, caspase 3, and poly ADP-ribose polymerase (PARP) was downregulated under gypenoside treatment. The specific mechanisms in vivo and in vitro of the above herbs are shown in Figure 3.The main findings of some studies that investigated the potential antioxidative stress effects of CHMs in atherosclerosis are summarized in Table 1.

## 4. Concluding Remarks and Future Directions

Through the literature research on atherosclerosis, we found that atherosclerosis is the pathological basis of many diseases and can damage many organs, especially the heart and brain [116, 117]. TCM is a traditional medical discipline established by ancient and modern physicians based on abundant clinical practice [118]. CHMs have the characteristics of multiple components and multiple targets [119], which can protect multiple organs caused by atherosclerosis. As an important mechanism for the occurrence and development of atherosclerosis, oxidative stress is a key target for the treatment of the disease. At the same time, a great number of studies have been proved that oxidative stress system is activated in atherosclerosis [120]. Therefore, this article focuses on the antioxidative stress mechanism of TCM intervention in atherosclerosis. Based on the existing evidence, the mechanisms of endogenous sources of ROS include NOX, NOS, mitochondrial dysfunction, and xanthine oxidase, which play an important role in the oxidative stress response of atherosclerosis [121]. ROS can further damage vascular endothelial cells by affecting cell relaxation and contraction, cell apoptosis and cell adhesion factor expression, and cell permeability [122].

According to published articles, CHMs have been found to play an important role in the treatment of atherosclerosis [123], and a large number of in vivo and in vitro experiments have proved the effectiveness of CHMs on oxidative stress. Nevertheless, research on the oxidative stress mechanism of CHMs intervention in atherosclerosis still leaves some key issues. First of all, past work has mostly focused on the expression of key molecules in the oxidative stress pathway of atherosclerosis and most of the studies on the pharmacological effects of CHM extracts are relatively isolated, leading to the unclear pharmacological effects of CHMs against oxidative stress. Therefore, more in-depth and extensive research is urgently needed in this area. Meanwhile, the majority of the research reports on the pharmacological effects of CHMs stays at the animal and cell level and rarely evaluate its clinical application. Thus, it is also necessary to explore the antioxidative stress mechanism of CHMs in the treatment of atherosclerosis in combination with clinical research. In addition, the characteristics of multicomponent TCM are a double-edged sword. On the one hand, atherosclerosis requires multitarget treatment. CHMs have the characteristics of multiple components, multiple targets, and multiple pathways and have little toxic and side effects, so it has its unique advantages in the treatment of complex diseases. On the other hand, the unclear active ingredients make it difficult to study the mechanism of CHM intervention in oxidative stress of atherosclerotic, which increases the difficulty of CHMs. With the development of systems biology, network pharmacology, and modern pharmacological research technology, we can apply these technologies to analyze and predict drug-target interactions, carrying out further experimental studies to explore the mechanism of CHMs in more depth. In a concise summary, we need to further explore the specific mechanism of CHMs for the treatment of atherosclerosis based on more complete clinical, animal and cell experiments, tapping the potential value of CHMs to provide a research basis for its better clinical application, and ultimately promote the modernization of CHMs in the treatment and prevention of CCVDs.

## Figures and Tables

**Table 1:** Detected studies reporting potential antioxidative stress effects of CHMs in atherosclerosis.

Reference	Author	Component	Experiment	Model	Relevant gene targets
[94]	J Shen	BYHWD	In vitro	HUVEC	NOX-4↓
[95]	J Guo	YXTM	In vitro	Sprague-Dawley rats	NF-*κ*B↓, MMP-9↓, Bcl-2↓, Caspase 3↑
[96]	B Wang	TXL	In vivo	C57BL/6 mice	VEGF↑, p-VEGFR2↑, p-PI3K↑, p-AKT↑, p-eNOS↑, HO-1↑, NOX-4↓
[97]	Y Ren	SMYAD	In vivo	C57BL/6 mice	SOD-1↑, SOD-2↑
[98]	K Guo	XJEK	In vivo	Wistar rats	P-JNK↓, P-p38MAPK↓, NOX-2↓
[99]	L Wang	BSNXD	In vitro	HUVEC	ER*β*↑, MDA↓, eNOS↑, NO↑, NF- *κ* B↓, MCP-1↓, ICAM-1↓, VCAM-1↓, E-selectin↓, CCR2 ↓, LFA-1 ↓, VLA-4 ↓
[100]	Y Yin	XMJ	In vitro	HUVEC	ICAM-1↓, VCAM-1↓, IL-1↓, IL-6↓
[101]	J Wen	TPHXR	In vivo	ApoE(-/-) mice	CAV-1↓, NF-*κ*B p50↓, NF-*κ*B p65↓, ICAM-1↓, VCAM-1↓, TNF-*α*↓, IL-6↓, IL-1*β*↓, hs-CRP↓, eNOS↑, NO↑
[102]	H Dong	Icariin	In vitro	Human brain vascular cerebrovascular smooth muscle cells	NOX-2↓
[103]	H Xu	Puerarin	In vitro	H9c2 cells	NOX-2↓
[104]	J Mo	Scutellarin	In vivo	Sprague-Dawley rats	NOX-4↓, SOD-1↑
[105]	Y. Li	Aucubin	In vitro	SH-SY5Y cells	TNF-*α*↓, IL-6↓, IL-1*β*↓, MDA↓, GSH↑, SOD↑, iNOS↓, NO↓
[106]	C. Shen	Bergaptol	In vitro	RAW264.7 cells	NO↓, IL-6↓, TNF-*α*↓, iNOS↓, IL-1*β*↓, COX-2↓, MAPK↓, NF-*κ*B↓, JNK↓, p38↓, p65↓, I*κ*B*α*↓, I*κ*K*α*/*β*↓, SRA1↓, CD-36↓
[107]	U Sundar	AEPS	In vitro	HUVEC	DDAH1↑, eNOS↑
[108]	Y. Yang	KRG-WE	In vitro	RAW264.7 cells	NO↓, iNOS↓, COX↓, IFN-*β*↓, p38↓, JNK↓, TBK1↓, ATF-2↓, CREB↓, IRF-3↓
[109]	N. Lian	Ginkgetin	In vivo	Sprague-Dawley rats	MMP-2↓, MMP-9↓, iNOS↓, NO↑, NOS↑
[110]	Y. Liu	Honokiol	In vivo	ApoE(-/-) mice	TNF-*α*↓, IL-6↓, IL-1*β*↓, NO↓, iNOS↓
[111]	Y. Luo	TASAESs	In vivo	ApoE(-/-) mice	Caspase 3↓
[112]	Q. Zhou	OPCRR	In vivo	Wistar rats	LDL-C↓, ox-LDL↓, HDL-C↑, SOD ↑, GSH-Px↑, MDA↓, TNF-*α*↓, IL-1*β*↓, IL-6↓, ICAM-1↓, VCAM-1↓, IL-10↑, NO↓, iNOS↓, eNOS↑
[113]	Z Peng	13-MB	In vitro	HUVEC	IL-1*β*↓, NLRP3↓, Caspase 1↑
[114]	Q Xu	TMP	In vitro	HUVEC	PGC-1*α*↑, NRF1↑, TFAM↑, SIRT1↑
[115]	N Song	Gypenoside	In vitro	EA. hy926 cells	PI3K↑ AKT↑, Bad↑, Cyt-c↓, Caspase 9↓, Caspase 3↓, PARP↓
[114]	Q Xu	TMP	In vivo	Sprague-Dawley rats	PGC-1*α*↑, NRF1↑, TFAM↑, SIRT1↑
[115]	N Song	Gypenoside	In vivo	ApoE(-/-) mice	PI3K↑, AKT↑, Bad↑, Cyt-c↓, Caspase 9↓, Caspase 3↓, PARP↓
